# Myeloid Angiotensin II Type 1 Receptor Mediates Macrophage Polarization and Promotes Vascular Injury in DOCA/Salt Hypertensive Mice

**DOI:** 10.3389/fphar.2022.879693

**Published:** 2022-06-03

**Authors:** Xue-Feng Yang, Huan Wang, Yue Huang, Jian-Hua Huang, Hao-Lin Ren, Qian Xu, Xiao-Min Su, Ai-Mei Wang, Fu Ren, Ming-Sheng Zhou

**Affiliations:** ^1^ Department of Physiology, Jinzhou Medical University, Jinzhou, China; ^2^ Department of Physiology, Shenyang Medical College, Shenyang, China; ^3^ The First Affiliated Hospital, Jinzhou Medical University, Jinzhou, China; ^4^ Radiology Department of the First Affiliated Hospital, Dalian Medical University, Dalian, China; ^5^ Department of Anatomy, Shenyang Medical College, Shenyang, China

**Keywords:** Ang II type 1 receptor, endothelial dysfunction, macrophage phenotype, salt-sensitive hypertension, vascular injury

## Abstract

Activation of the renin–angiotensin system has been implicated in hypertension. Angiotensin (Ang) II is a potent proinflammatory mediator. The present study investigated the role of myeloid angiotensin type 1 receptor (AT1R) in control of macrophage phenotype *in vitro* and vascular injury in deoxycorticosterone acetate (DOCA)/salt hypertension. In human THP-1/macrophages, Ang II increased mRNA expressions of M1 cytokines and decreased M2 cytokine expressions. Overexpression of AT1R further increased Ang II-induced expressions of M1 cytokines and decreased M2 cytokines. Silenced AT1R reversed Ang II-induced changes in M1 and M2 cytokines. Ang II upregulated hypoxia-inducible factor (HIF)1α, toll-like receptor (TLR)4, and the ratio of pI*κ*B/I*κ*B, which were prevented by silenced AT1R. Silenced HIF1α prevented Ang II activation of the TLR4/NF*κ*B pathway. Furthermore, Ang II increased HIF1α *via* reactive oxygen species-dependent reduction in prolyl hydroxylase domain protein 2 (PHD2) expression. The expressions of AT1R and HIF1α and the ratio of pI*κ*B/I*κ*B were upregulated in the peritoneal macrophages of DOCA hypertensive mice, and the specific deletion of myeloid AT1R attenuated cardiac and vascular injury and vascular oxidative stress, reduced the recruitment of macrophages and M1 cytokine expressions, and improved endothelial function without significant reduction in blood pressure. Our results demonstrate that Ang II/AT1R controls the macrophage phenotype *via* stimulating the HIF1α/NF*κ*B pathway, and specific myeloid AT1R KO improves endothelial function, vascular inflammation, and injury in salt-sensitive hypertension. The results support the notion that myeloid AT1R plays an important role in the regulation of the macrophage phenotype, and dysfunction of this receptor may promote vascular dysfunction and injury in salt-sensitive hypertension.

## Introduction

Inappropriate activation of the renin–angiotensin system (RAS) is associated with increased cardiovascular inflammation and risk of hypertensive and cardiovascular diseases (Li et al., 2008). Angiotensin (Ang) II is a main component of the RAS and plays an important role in the regulation of blood pressure and water and sodium balance (Szczepanska-Sadowska et al., 2018). Ang II exerts its biological effects mainly by binding to angiotensin type 1 receptor (AT1R). AT1R blockers (ARBs) have been proved to be beneficial to the patients with hypertension and hypertensive animal models (Abraham et al., 2015; Omboni and Volpe, 2019). Although it is generally accepted that the therapeutic effects of ARBs on hypertension and cardiovascular diseases are achieved mainly by directly blocking AT1R in the target of cardiovascular tissues (Akazawa et al., 2013), AT1R is widely distributed in other cells or tissues, such as immune cells, brain cells, or skeletal muscle cells (Marino et al., 2008; Zhou et al., 2015; Haspula and Clark, 2018; Deminice et al., 2020). The contribution of AT1R in other cells, especially in immune cells, to hypertension and hypertensive end-organ damage is still poorly understood.

Ang II is a proinflammatory mediator. Ang II promotes macrophage accumulation in the infiltrated tissues and release of proinflammatory cytokines (Maranduca et al., 2020), and AT1R antagonists reduce vascular inflammation and macrophage infiltration in hypertensive animal models. Macrophages have remarkable phenotypic heterogeneity and plasticity, and the phenotype spectrum of macrophages can be characterized, at the extremes, by the classically activated M1 macrophages and the alternatively activated M2 macrophages (Italiani and Boraschi, 2014). The classically activated M1 macrophages are usually produced during cell-mediated host immune response and typically induced by lipopolysaccharide (LPS) or Th1 cytokines, such as tumor necrosis factor (TNF)α and interferon (IFN)γ (Zhang et al., 2012; Dungan et al., 2014; Orecchioni et al., 2019). M1 macrophages are more susceptible to the migration of the vascular wall and the release of inflammatory cytokines, such as TNFα and interleukin (IL)-1β, which may initiate vascular inflammation, oxidative stress, and vascular damage (Cai et al., 2020).

Salt sensitivity (SS) affects about 50% of hypertensive patients. SS hypertension is traditionally considered to be “volume-dependent hypertension.” Due to the low level of plasma renin, the role of Ang II is presumed to be irrelevant. However, our and other studies on Dahl SS (DS) rats, a paradigm of low-renin SS hypertension in humans, have shown that SS hypertension is accompanied by the upregulation of the local tissue angiotensin system (Zhou et al., 2003, 2006). The blockade of AT1R with candesartan improves endothelial function and vascular insulin signaling and reduces vascular oxidative stress and inflammation without significant reduction in blood pressure (Zhou et al., 2003, 2009). The chemical depletion of macrophages with liposome-encapsulated chlodronate can produce similar vascular beneficial effects and significantly reduce vascular inflammation and macrophage infiltration in hypertensive Dahl rats. These studies suggest that despite the low plasma renin level, the upregulation of the local Ang II system may play an important role in the pathogenesis of SS hypertension (Narumi et al., 2015). Macrophages express all components of RAS, including AT1R (Moratal et al., 2021). However, it is unclear whether Ang II/AT1R plays a role in the regulation of macrophage function and whether macrophage Ang II/AT1R signaling is involved in the pathogenesis of low renin SS hypertension. In the present study, we investigated the role of AT1R in the regulation of the macrophage phenotype *in vitro* and endothelial function and cardiovascular injury in deoxycorticosterone acetate (DOCA)/salt hypertensive mice.

## Materials and Methods

### Cell Culture

Human THP-1 monocytes (TIB-202) were purchased from ATCC (Manassas, VA) and cultured in RPMI 1640 medium supplemented with 10% fetal bovine serum (FBS), 100 U/ml penicillin, and 100 μg/ml streptomycin. The cells were grown at 37°C and 95% humidified air with 5% CO_2_ and used between passages 4 to 16. THP-1 monocytes (5 × 10^5^ cells/ml) were seeded in six-well dishes and incubated with phorbol 12-myristate 13-acetate (PMA, 100 ng/ml) for 48 h. Then, THP-1 cells were adherent to the wall and differentiated into macrophages. THP-1 macrophages were starved in serum-free RPMI 1640 medium for 24 h before the experiments were performed.

### Cell Transfection

The AT1R cloned overexpression plasmid and negative control vector were purchased from Sino Biological Inc. (Beijing, China). AT1R transfection was performed using Lipofectamine 2000 according to the manufacturer’s instructions. In brief, a total of 1 μg AT1R plasmid or control vector with lipofectamine 2000 was added to serum- and antibiotic-free 1640 medium. After mixing, the medium was incubated at room temperature for 5 min and added to cultured macrophages. The cells were harvested at 48 h after transfection. The efficiency of AT1R transfection was confirmed by a more than 3-fold increase in AT1R mRNA expression compared with the control vector.

siRNA transfections for AT1R or hypoxia-inducible factor (HIF)1α were performed using Lipofectamine 2000. siRNA AT1R, siRNA HIF1α, and siRNA control (scrabbled siRNA) were purchased from Santa Cruz Biotechnology. The macrophages were resuspended with trypsin and transfected using siRNA AT1R or siRNA HIF1α (25 nM, 50 nM and 100 nM) in serum- and antibiotic-free transfection medium. Then the cells were incubated for 48 h according to the manufacturer’s instructions (Santa Cruz Biotechnology). The efficiency of siRNA transfection was confirmed by more than 70% reduction in the target mRNA by real-time PCR, compared with the siRNA control group.

### Peritoneal Macrophage Isolation and Culture

Another group of mice was used to isolate peritoneal macrophages as described previously (Liu et al., 2017). The mice were intraperitoneally (I.P) injected with 0.5 ml of a 3% thioglycollate medium. On day 5 of post-injection, the mice were anesthetized with sodium pentobarbital (50 mg/kg I.P), the abdominal cavity was washed with about 1–2 ml cold PBS containing 10 mmol/L EDTA, and the peritoneal exudate was collected and centrifuged at 400 g for 10 min. The cell pellets were washed twice with PBS and then resuspended in 0.2 ml DMEM to count the cells. The isolated macrophages were plated separately in 6-well tissue culture plates and cultured with RPMI/10% FBS overnight. The next morning, nonadherent cells were removed by aspiration, and adherent cells (macrophages) were washed with PBS three times and harvested for the experiments.

### Real-Time PCR

Cells were harvested in 1 ml TRIzol reagent (Takara, Japan), and RNA was isolated and quantified using a Nanodrop spectrophotometer (Wilmington, United States). Total RNA of 2 μg was reversely transcribed using AMV reverse transcriptase and random primers (Takara, Japan) according to the manufacturer’s instructions. cDNA amplification was performed using the SYBR Premix Ex Taq II kit (Takara, Japan). The relative quantities of each transcript were normalized by a housekeeping gene of GAPDH, and PCR primers for all target genes, including AT1R, TNFα, IL-1β, inducible nitric oxide synthase (iNOS), arginase, and IL4 for THP1 macrophages, were designed according to the sequences in GenBank, the primer gene sequences are summarized in Table 1. Ang II was purchased from Sigma-Aldrich (cat. #: A9525).

**TABLE 1:** Primer sequences (THP-1 cells).

Name	Primer sequence	AT (°C)	Product size (bp)	Extension time (s)
AT1R	F: 5′-GCC​AGT​TTG​CCA​GCT​ATA​ATC​C-3′	60	200	39
	R: 5′-GCC​TTC​TTT​AGG​GCC​TTC​CA-3′			
TNFα	F: 5′-ACT​TTG​GAG​TGA​TCG​GCC​CC-3′	60	193	39
	R: 5′-CCA​TTG​GCC​AGG​AGG​GCA​TT-3′			
IL-1β	F: 5′-GAA​GCT​GAT​GGC​CCT​AAA​CA-3′	60	110	39
	R: 5′-AAG​CCC​TTG​CTG​TAG​TGG​TG-3′			
iNOS	F: 5′-GGA​ACC​TAC​CAA​CTG​ACG​GG-3′	60	175	39
	R: 5′-TGG​AGT​AAC​GCA​CGT​GTC​TG-3′			
Arginase	F: 5′-GCC​AAG​TCC​AGA​ACC​ATA​GG-3′	60	105	39
	R: 5′-AAG​CAG​ACC​AGC​CTT​TCT​CA-3′			
IL4	F: 5′-CAG​TTC​TAC​AGC​CAC​CAT-3′	60	159	39
	R: 5′-CTG​GTT​GGC​TTC​CTT​CAC-3′			
GAPDH	F: 5′-ACC​ACA​GTC​CAT​GCC​ATC​AC-3′	60	147	39
	R: 5′-CAG​CTC​AGG​GAT​GAC​CTT​GC-3′			

AT, annealing temperature.

### Western Blot Analysis

THP-1 macrophages, peritoneal macrophages or aortic tissues were homogenized in RIPA lysis buffer containing the cocktail of protease inhibitor mixture. After homogenization, an aliquot of the supernatant was used for the protein measurement with Bio-Rad protein assay. An equal amount (30 μg) of protein was separated by SDS-PAGE and transferred to a polyvinylidene fluoride membrane (PVDF). The membrane was blocked with 5% skim milk in Tris-buffered saline with Tween 20 at room temperature for 1 h. The membrane was then incubated overnight at 4°C with the primary antibodies against HIF1α (Santa Cruz Biotech., 1:500 dilution), TLR4 (Santa Cruz Biotech., 1:500 dilution), inhibitory kappa B (I*κ*B, Santa Cruz Biotech., 1:500 dilution), pI*κ*B (ser 32, Santa Cruz Biotech., 1:500 dilution), TNFα (Wanleibio, 1:500 dilution), IL-1β (Wanleibio, 1:1,000 dilution), prolyl hydroxylase domain protein (PHD)2 (Santa Cruz Biotech., 1:500 dilution), phospho-endothelial nitric oxide synthase (p-eNOS, ser 1,177, Santa Cruz Biotech., 1:500 dilution), eNOS (Cell signaling Inc., 1:1,000 dilution). Then, the membrane was incubated with horseradish peroxidase-conjugated appropriate secondary antibody for 1 h at room temperature. The membrane was reblotted for GAPDH (Proteintech, 1:5,000 dilution) to serve as a loading control. The labeled proteins were detected using the Bio-Rad system and quantified through ImageJ software. The data were normalized to GAPDH and expressed as a fold increase versus the control group.

### Determination of Superoxide Anion (O_2_^−^) Production in THP-1/Macrophage

O_2_^−^ production in THP-1-derived macrophages was determined by lucigenin-enhanced chemiluminescence. In brief, macrophages (1 × 10^5^ cells/ml) were incubated with Ang II (100 nmol/L, Sigma-Aldrich, St. Louis, MO) for 24 h. In some experiments, the cells were preincubated with NADPH oxidase inhibitor diphenyleneiodonium (DPI, 10 μmol/L, Sigma-Aldrich, St. Louis, MO) or apocynin (100 μmol/L, Sigma-Aldrich, St. Louis, MO) for 30 min. The cells were added to a cuvette containing 1 ml of phosphate buffer saline (pH 7.4). The reaction was triggered by adding 5 μmol/L lucigenin. A buffer blank was subtracted from each reading. The results were adjusted according to the protein content and expressed as counts/min/mg protein.

### Animal Protocols

All animal protocols followed the international standard guide for the care and use of laboratory animals and were approved by the Institutional Animal Care and Use Committee of Shenyang Medical College. The mice that carried floxed AT1R alleles (LoxP sites flanking exons of AT1R gene, created by Shanghai Model Organism Center Inc.) were crossed with the mice that expressed Cre-recombinase in myeloid cells (Lyz2-Cre, provided by Shanghai Model Organism Center Inc.) to generate the mice with specific myeloid cell deficiency of AT1R (MyeAT1R^−/-^). Littermates of the MyeAT1R knockout (AT1R^loxp/loxp^) mice, which are of the same background as C57BL/6J wild-type mice, were used as knockout control (AT1R^ctr.^). All mice were genotyped before they were used for the experiments. To induce DOCA/salt hypertension, mice were anesthetized with 100 mg/kg ketamine and 20 mg/kg xylazine cocktail, and right nephrectomy was performed. A DOCA pellet (200 mg/60-day release, Innovative Research of American, Sarasota, FL) was implanted in the scapular region of mice. DOCA mice drank a solution containing 1% NaCl and 0.2% KCl. The control mice received sham operation and drank tap water. AT1R^loxp/loxp^ and MyeAT1R^−/-^ mice were divided into four groups and received one of the following treatments for five weeks: 1) Knockout control group (ctrl, N = 8): AT1R^loxp/loxp^ mice with sham surgery; 2) DOCA/salt hypertension group (DOCA, N = 8): AT1R^loxp/loxp^ with a right nephrectomy and the implantation with DOCA pellet; 3) MyeAT1R^−/-^ group (AT1R^−/-^, N = 8): MyeAT1R^−/-^ mice with sham surgery; 4) MyeAT1R^−/-^ with DOCA/salt hypertension group (DOCA/AT1R^−/-^, N = 8): MyeAT1R^−/-^ mice with a right nephrectomy and DOCA pellet implantation. Systolic blood pressure (SBP) was measured in the conscious mice using the tail-cuff method (Softron Blood Pressure Meter, BP-2010 Serial, Beijing). The mice were daily trained for five consecutive days to adapt to blood pressure measurement. SBP was measured on the day before surgery and DOCA pellet implantation, and thereafter once a week until the end of the experiment. At least five successive readings were taken, and the average value was taken as one measurement. At the end of the study, mice were euthanized by excessive anesthetic (sodium pentobarbital 100 mg/kg, I.P), and the heart and the aorta were harvested.

### Histological Studies

The thoracic aorta or a piece of the left ventricle (free wall) was fixed in 4% paraformaldehyde in phosphate-buffered saline and embedded in paraffin. The specimen was cut into 4 μm thick sections. The sections were stained with hematoxylin and eosin (HE, Sigma-Aldrich, St. Louis, MO). Four images from four nonconsecutive slides per sample were acquired using a digital camera, and the thickness of the aortic wall was measured and analyzed using the Image-Pro Plus version 6.0 software system. The cross-sectional areas of 100 cardiomyocytes in 4 randomly selected areas per slide were measured using a quantitative digital image analysis system (Media Cybernetics, Rockville) to assess cardiomyocyte hypertrophy. The sections were stained with Masson-Trichrome (Sigma-Aldrich, St. Louis, MO) to evaluate cardiac fibrosis. Eight randomly selected areas in two nonconsecutive slides per sample were examined to evaluate the semi-quantitative content of collagen, the percentage of positively stained areas to total areas of cardiac tissue was calculated using the Image-Pro Plus image analysis system. The histologic samples were blind to the reviewers who were not aware of the groups to which the mice belonged.

### Determination of Aortic O_2_^−^ Production *in situ*

Oxidative fluorescent dye hydroethidine (DHE, Sigma-Aldrich, St Louis, MO) was used for the determination of aortic O_2_^−^ production as previously described (Zhou et al., 2003). In brief, the thoracic aorta was embedded in the OCT compound with a snap frozen in liquid nitrogen. The samples were cut into 4-μm thick sections with a freezing microtome. The slides were incubated with 2 μmol/L DHE in HEPES buffer at 37°C for 30 min. The images in eight randomly selected fields in two nonconsecutive slides per sample were acquired by a confocal fluorescence microscope (Leica Microsystems Inc., Mannheim, Germany). Average fluorescent intensity was measured and used for the quantification of O_2_^−^ production.

### Organ Chamber Experiments

Endothelium-dependent vasorelaxation to acetylcholine or endothelium-independent relaxation to sodium nitroprusside was determined using an organ bath chamber (DMT Inc., Denmark) as previously described (Cai et al., 2020). The thoracic aorta was cut into 3-mm aortic rings, and the aortic rings were contracted twice with KPSS solution containing 60 mmol/L KCL. The rings were precontracted to 70% of maximal constriction force to norepinephrine (about 30 nmol/L norepinephrine, Sigma-Aldrich, St. Louis, MO). After the contraction reached a plateau, an accumulative dose of acetylcholine (10^–9^ to 10^–5^ mol/L, Sigma-Aldrich, St. Louis, MO) or sodium nitroprusside (10^–9^ to 10^–5^ mol/L, Sigma-Aldrich) was added in the organ chamber, and maximal response to an agonist (Emax) and the concentration of agonist required for a half-maximal response curve (ED_50_) were determined and calculated from the concentration-response curve, using best fit to a logistic sigmoid function.

### Immunofluorescence Analysis

The paraffin-embedded aortic tissues were cut into a 4 μm thick section and fixed on the slides. The slides were placed in the microwave at 60°C for 1 h for antigen retrieval and then incubated with a blocking solution of 5% bovine serum albumin at room temperature for 1 h. The sections were incubated with the primary antibody against F4/80 (Cell Signaling Technology, Rabbit mAb #30325, 1:400 dilution) at 4°C overnight, and then the sections were incubated with the secondary antibody (red) of donkey anti-rabbit IgG (Abcam, H&L, ab150075 Alexa Fluor 647, 1:1,000 dilution) at room temperature for 1h. The nuclei were stained with DAPI. F4/80 positive cells (monocyte/macrophage) were viewed by fluorescence microscope. At least five images per slide were examined and averaged as a single value, and the number of positive F4/80 staining cells was counted and expressed as per μm^2^ area of the selected view area.

### Statistical Analysis

Statistical analysis was carried out using SPSS 18.0 software for Windows (SPSS Inc., Chicago, IL, United States). All experimental data were expressed as mean ± standard error of the mean (SEM). The data were analyzed with one-way or two-way ANOVA followed by Tukey’s test for multiple corrections between groups. The sample size and statistical method for post-analysis were described in each figure legend. The values were considered significant when *p* < 0.05.

## Results

### Ang II Controls Macrophage Phenotype and Polarization Through AT1R

**FIGURE 1:**
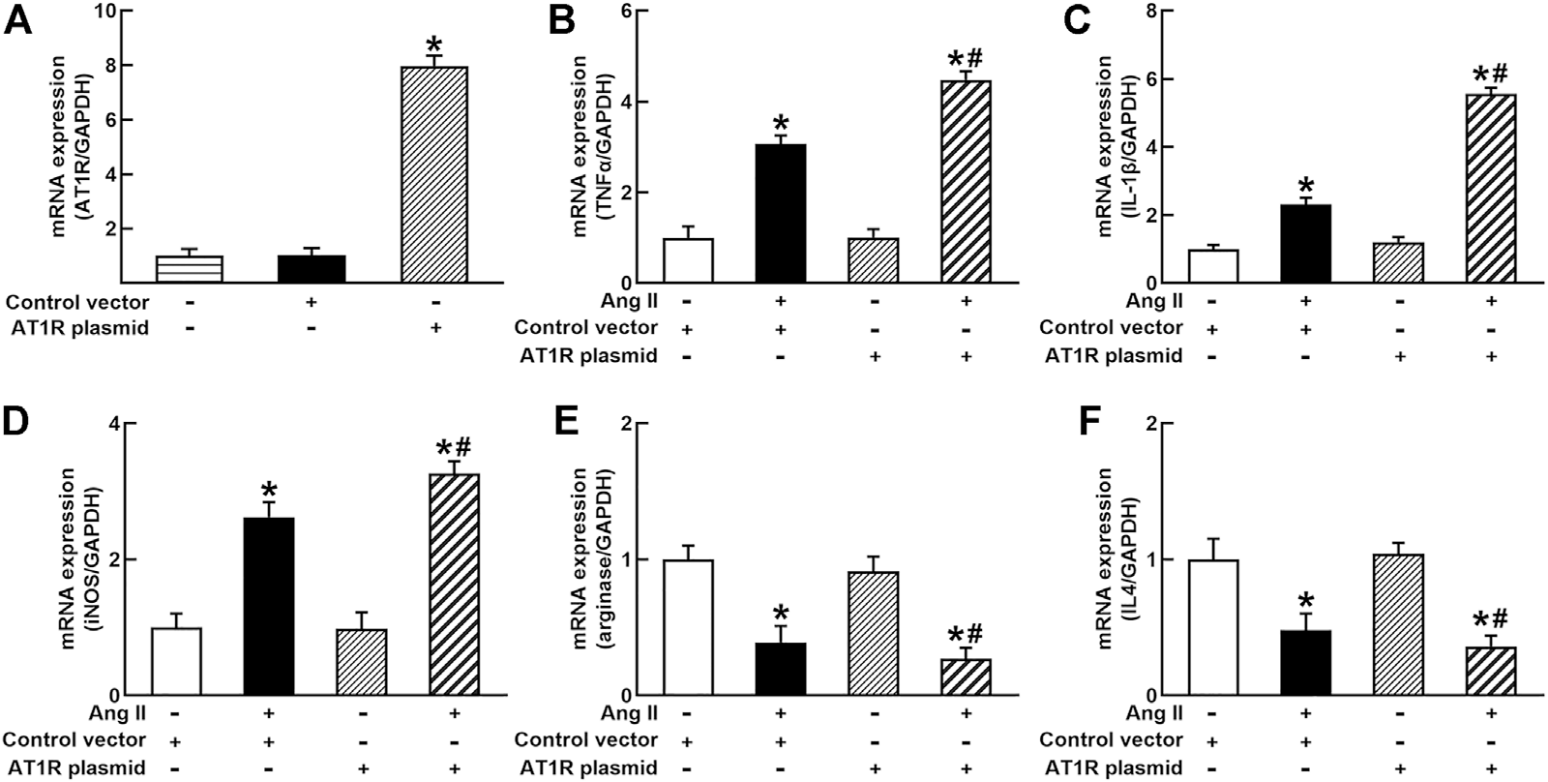
Effects of angiotensin type 1 receptor (AT1R) overexpression on mRNA expressions of M1 and M2 macrophage cytokines in THP-1/macrophages. The transfection of the *AT1R* gene increased AT1R expression **(A)**; AT1R overexpression potentiated Ang II-induced mRNA expression of M1 macrophage cytokines such as tumor necrosis factor (TNFα) **(B)**, interleukin 1β (IL-1β) **(C)**, and inducible nitric oxide synthase (iNOS) **(D)**; overexpression of AT1R further decreased Ang II-induced mRNA expression of M2 macrophage cytokine genes arginase **(E)** and IL4 **(F)**. Statistical comparisons were carried out with one-way ANOVA **(A)** or two-way ANOVA **(B–F)** followed by the Tukey post hoc test. Data were expressed as mean ± SEM. N = 6, *p < 0.05 vs. control vector, #*p* < 0.05 vs. Ang II group.

**FIGURE 2:**
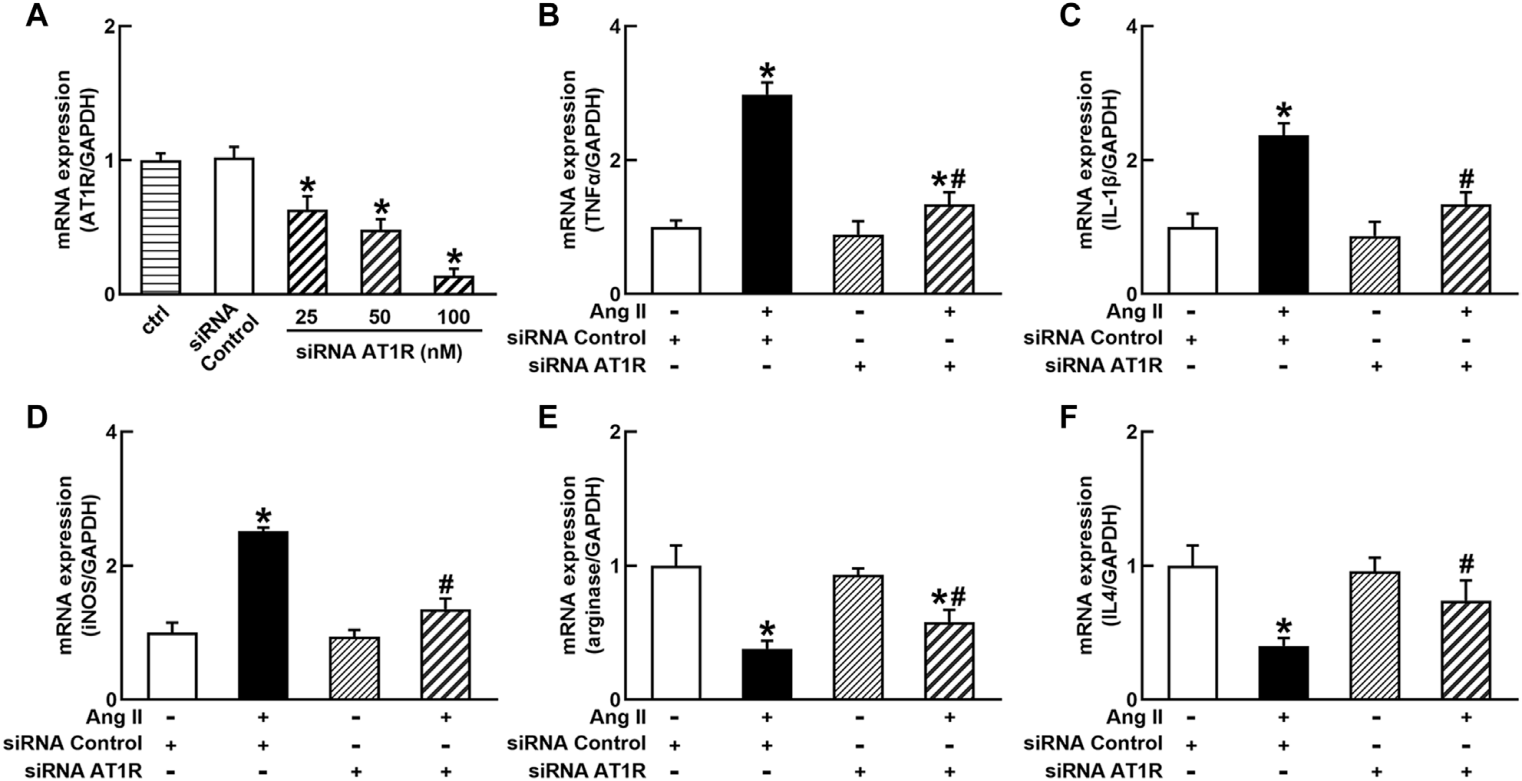
Effects of silenced AT1R on the mRNA expressions of M1 and M2 macrophage cytokines in THP-1/macrophages. siRNA AT1R dose dependently reduced mRNA expression of AT1R **(A)**; silenced *AT1R* gene prevented Ang II-induced increase in M1 cytokines TNFα **(B)**, IL-1β **(C)**, and iNOS **(D)**; silenced AT1R prevented Ang II-induced decrease in mRNA expressions of M2 cytokines, arginase **(E)** and IL4 **(F)**. Statistical comparisons were carried out with one-way ANOVA **(A)** or two-way ANOVA **(B–F)** followed by the Tukey post hoc test. Data were expressed as mean ± SEM. N = 6, *p < 0.05 vs. siRNA control, #*p* < 0.05 vs. Ang II group.

To identify the role of AT1R in the control of macrophage polarization, we transfected an AT1R clone plasmid (AT1R plasmid) or negative control vector (control vector) to THP-1/macrophages, the transfection of AT1R plasmid resulted in an 8-fold increase in mRNA expression of AT1R but control vector did not affect mRNA expression of AT1R (Figure 1A). Treatment with Ang II (100 nmol/L) increased mRNA expressions of M1 macrophage proinflammatory cytokines, including TNFα, IL-1β, and iNOS, and the overexpression of the AT1R gene further enhanced the expressions of above M1 macrophage cytokines stimulated by Ang II (Figures 1B–D). Ang II decreased mRNA expressions of M2 macrophage markers arginase and IL4. Overexpression of AT1R further decreased the expression of Ang II-induced arginase and IL4 (Figures 1E and F). In another way, we silenced the *AT1R* gene using siRNA AT1R. As shown in Figure 2A, siRNA AT1R dose-dependently reduced mRNA expression of AT1R in macrophages, with a maximal reduction (80%) at 100 nmol/L siRNA AT1R. This concentration of siRNA (100 nmol/L) was selected for siRNA experiments, and siRNA AT1R reversed the expressions of above M1 or M2 cytokines induced by Ang II (Figures 2B–F). These results suggest that Ang II regulates the activation and phenotype of macrophages *via* activating AT1R.

### Ang II/AT1R Induces Macrophage Activation by Stimulating the HIF1α/NFκB Pathway

**FIGURE 3:**
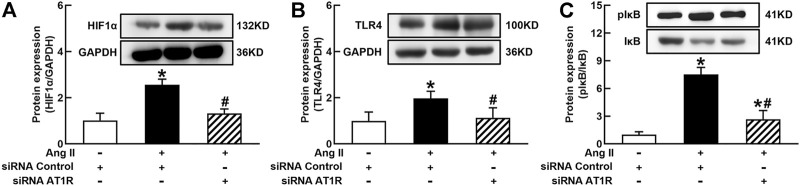
Silenced AT1R prevented Ang II activation of hypoxia-induced factor (HIF)1α/toll-like receptor (TLR4)/nuclear factor (NF)*κ*B pathway in THP-1/macrophages. Silenced *AT1R* gene prevented Ang II-induced increase in protein expression of HIF1α **(A)**, TLR4 **(B)**, and the ratio of pI*κ*B/I*κ*B **(C)**. Statistical comparisons were carried out with two-way ANOVA followed by the Tukey post hoc test. Data were expressed as mean ± SEM. N = 6, *p < 0.05 vs. siRNA control, #*p* < 0.05 vs. Ang II group.

**FIGURE 4:**
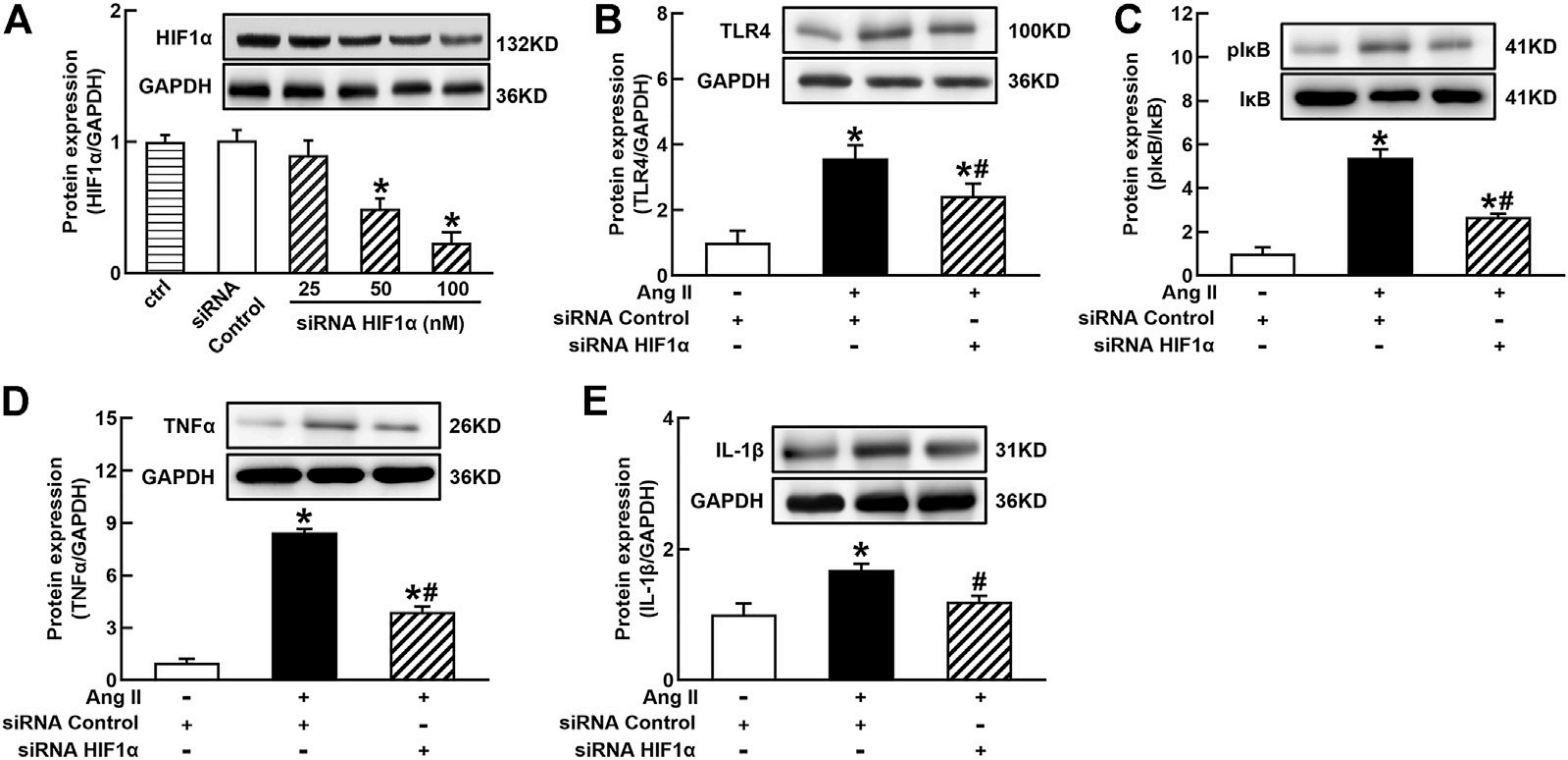
Silenced HIF1α inhibited Ang II-induced TLR4/NF*κ*B activation and inflammatory cytokine expressions in THP-1/macrophages. siRNA HIF1α dose dependently decreased protein expression of HIF1α **(A)** and reduced Ang II-induced increase in TLR4 **(B)**, the ratio of pI*κ*B/I*κ*B **(C)**, and proinflammatory cytokines, TNFα **(D)** and IL-1β **(E)**. Statistical comparisons were carried out with one-way ANOVA **(A)** or two-way ANOVA **(B–E)** followed by the Tukey post hoc test. Data were expressed as mean ± SEM. N = 6, *p < 0.05 vs. siRNA control, #*p* < 0.05 vs. Ang II group.

It is well known that the innate immune response triggered by infection is mediated by the TLRs/NF*κ*B pathway, which is usually related to a hypoxic environment (Nishi et al., 2008). HIF1α is a transcriptional factor which controls hypoxia-related gene expressions (Fang et al., 2009). The activation of HIF1α can change metabolic pathway and induces macrophage activation under a hypoxia environment (Kim et al., 2010). To elucidate the mechanisms of Ang II activation of macrophages, we investigated the effects of siRNA AT1R on HIF1α, TLR4, and NF*κ*B signaling pathways in Ang II-treated THP-1/macrophages. Ang II (100 nmol/L) significantly increased the protein expressions of HIF1α, TLR4, and the ratio of pI*κ*B/I*κ*B. Silenced AT1R gene prevented Ang II-induced increase in the expressions of HIF1α, TLR4, and the ratio of pI*κ*B/I*κ*B (Figures 3A–C). Next, we investigated the effects of siRNA HIF1α on the TLR4/NF*κ*B pathway in Ang II-treated macrophages. siRNA HIF1α dose dependently reduced the protein expression of HIF1α with a maximal reduction of 80% in 100 nmol/L siRNA HIF1α (Figure 4A). Silenced HIF1α significantly attenuated Ang II-induced increase in the expressions of TLR4 and the ratio of pI*κ*B/I*κ*B (Figures 4B and C). Furthermore, silenced HIF1α inhibited Ang II-induced expressions of proinflammatory cytokines TNFα and IL-1β (Figures 4D and E), and both TNFα and IL-1β are the downstream molecules of NF*κ*B. Thus, these results suggest that Ang II/AT1R induces macrophage activation *via* activating HIF1α/TLR4/NF*κ*B pathway.

### Ang II/AT1R Stimulates HIF1α Expression *via* the Generation of ROS

**FIGURE 5:**
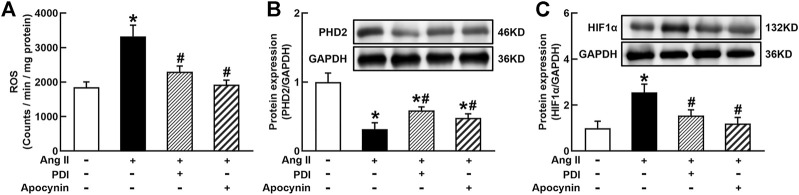
Antioxidant treatment inhibited Ang II-induced reactive oxygen species (ROS) production and HIF1α expression in THP-1/macrophages. Antioxidants reversed Ang II-induced ROS production **(A)**, inhibition of prolyl hydroxylase domain protein 2 (PHD2) **(B),** and upregulation of HIF1α expression **(C)**. Statistical comparisons were carried out with one-way ANOVA followed by the Tukey post hoc test. Data were expressed as mean ± SEM. N = 6, *p < 0.05 vs. control, #*p* < 0.05 vs. Ang II group.

It is well known that Ang II increases ROS generation *via* binding to AT1R in vascular cells and many other types of cells (Zhou et al., 2009, 2015). ROS can stabilize HIF1α *via* the degradation of PHD2 because PHD2 can hydroxylate and promote HIF1α degradation during normal oxygen conditions (Poblete et al., 2020). As shown in Figure 5A, Ang II significantly increased O_2_^−^ production in THP-1/macrophage, treatment with NADPH oxidase inhibitors DPI or apocynin inhibited Ang II-induced O_2_^−^ production. Ang II (100 nmol/L) decreased PHD2 and increased HIF1α expression. Antioxidants DPI or apocynin reversed Ang II-induced changes in PHD2 and HIF1α expressions (Figures 5B and C). These results suggest that Ang II can stabilize and induce HIF1α activation through ROS inhibition of PHD2-mediated HIF1α degradation.

### Specific Knockout of Myeloid *AT1R* Gene Reduced the Expressions of AT1R, HIF1α, and NF*κ*B Activation in the Peritoneal Macrophages of DOCA Hypertensive Mice

**FIGURE 6:**
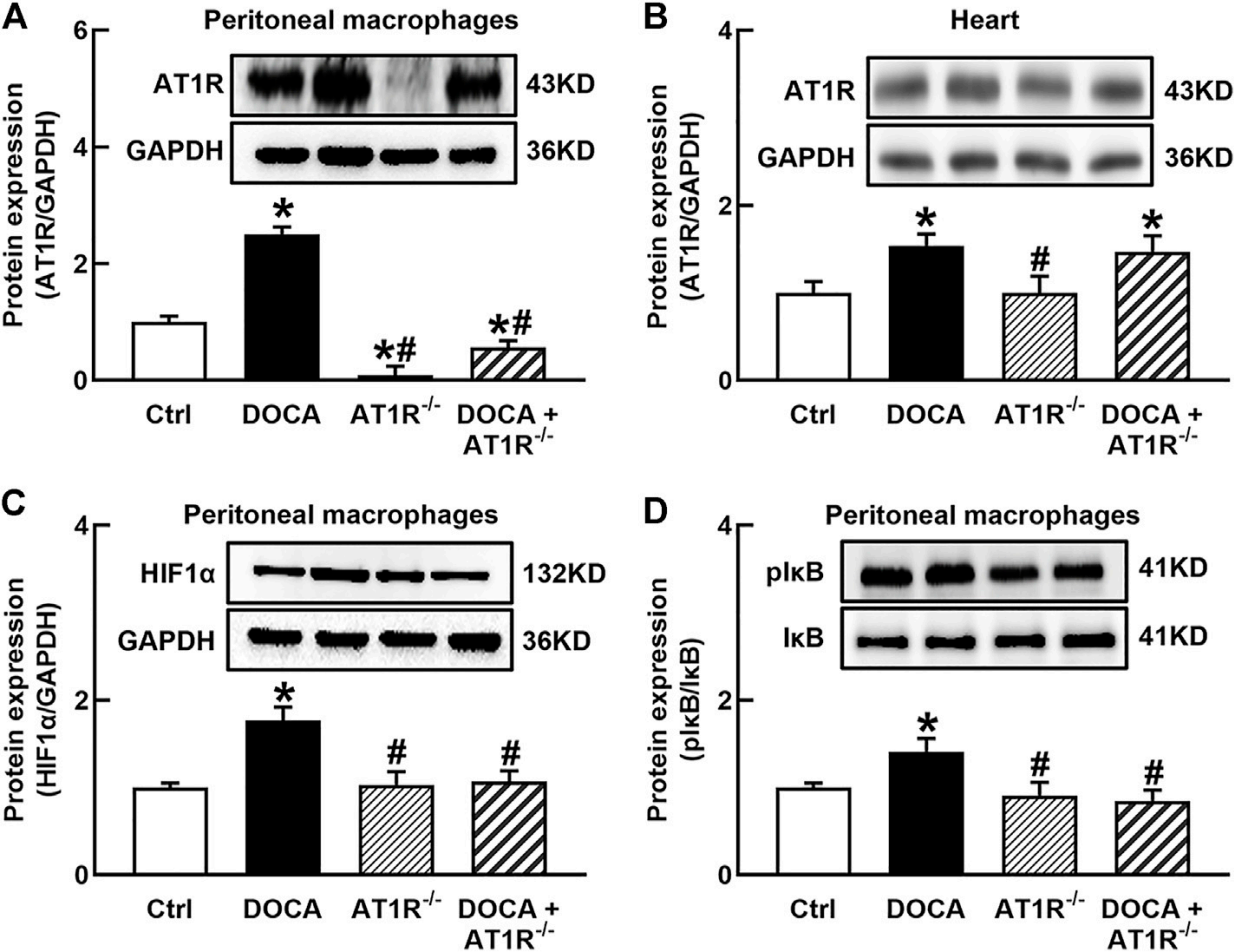
Specific myeloid AT1R KO reduced the expression of AT1R **(A)**, HIF1α**(C)**, and the ratio of pI*κ*B/I*κ*B **(D)** in the peritoneal macrophage of DOCA mice. Myeloid AT1R KO did not significantly affect AT1R expression in the heart **(B)**. Statistical comparisons were carried out with two-way ANOVA followed by the Tukey post hoc test. Data were expressed as mean ± SEM. N = 6, *p < 0.05 vs. control mice, #*p* < 0.05 vs. DOCA mice.

To investigate the role of macrophage AT1R in SS hypertension, we generated specific MyeAT1R^−/-^ mice by crossing AT1R^loxp/loxp^ mice with the Lyz2-Cre mice. The mice were treated with DOCA and salt to induce DOCA SS hypertension. There were no significant differences in body weight and heart rate among normal control, AT1R KO, and hypertensive DOCA mice (Table 2). There was no significant difference in plasma levels of Na^+^, K^+^, and Cl^−^ between normal control mice and DOCA hypertensive mice. AT1R KO did not affect plasma Na^+^, K^+^ and Cl^−^ levels in normal control and hypertensive mice (Table 2). As shown in Figure 6A, the protein expression of AT1R was significantly increased in the peritoneal macrophages of DOCA hypertensive mice. Specific KO of myeloid AT1R significantly reduced more than 80% of AT1R protein expression from the peritoneal macrophages in either control or hypertensive mice. AT1R expression was also increased in the heart tissue of DOCA hypertensive mice, but the knockout of the myeloid AT1R gene did not affect heart AT1R expression (Figure 6B), confirming that MyeAT1R^−/-^ mice have a specific target of AT1R in myeloid cells. The expressions of HIF1α and the ratio of pI*κ*B/I*κ*B were increased in the peritoneal macrophage of DOCA hypertensive mice, and the depletion of myeloid AT1R prevented HIF1α expression and NF*κ*B activation in DOCA hypertensive mice (Figures 6C and D).

**TABLE 2:** Effects of AT1R^−/-^ on BW, HR, and plasma electrolyte in DOCA-salt mice.

	Ctrl	DOCA	AT1R^−/-^	DOCA+AT1R^−/-^
BW (g)	24.3 ± 1.3	22.8 ± 1.2	24.9 ± 1.3	23.9 + 1.4
HR (beats/min)	625 ± 17	622 ± 11	627 ± 16	626 ± 12
Na^+^ plasma (mM)	145 ± 4	147 ± 5	143 ± 5	147 ± 3
K^+^ plasma (mM)	4.3 ± 0.2	4.1 ± 0.3	4.2 ± 0.2	4.2 ± 0.3
Cl^−^ plasma (mM)	108 ± 3	106 ± 4	107 ± 4	107 ± 3

### Myeloid AT1R Deficiency Protects Against Cardiovascular Injury Without Significantly Reducing SBP in DOCA SS Mice

**FIGURE 7:**
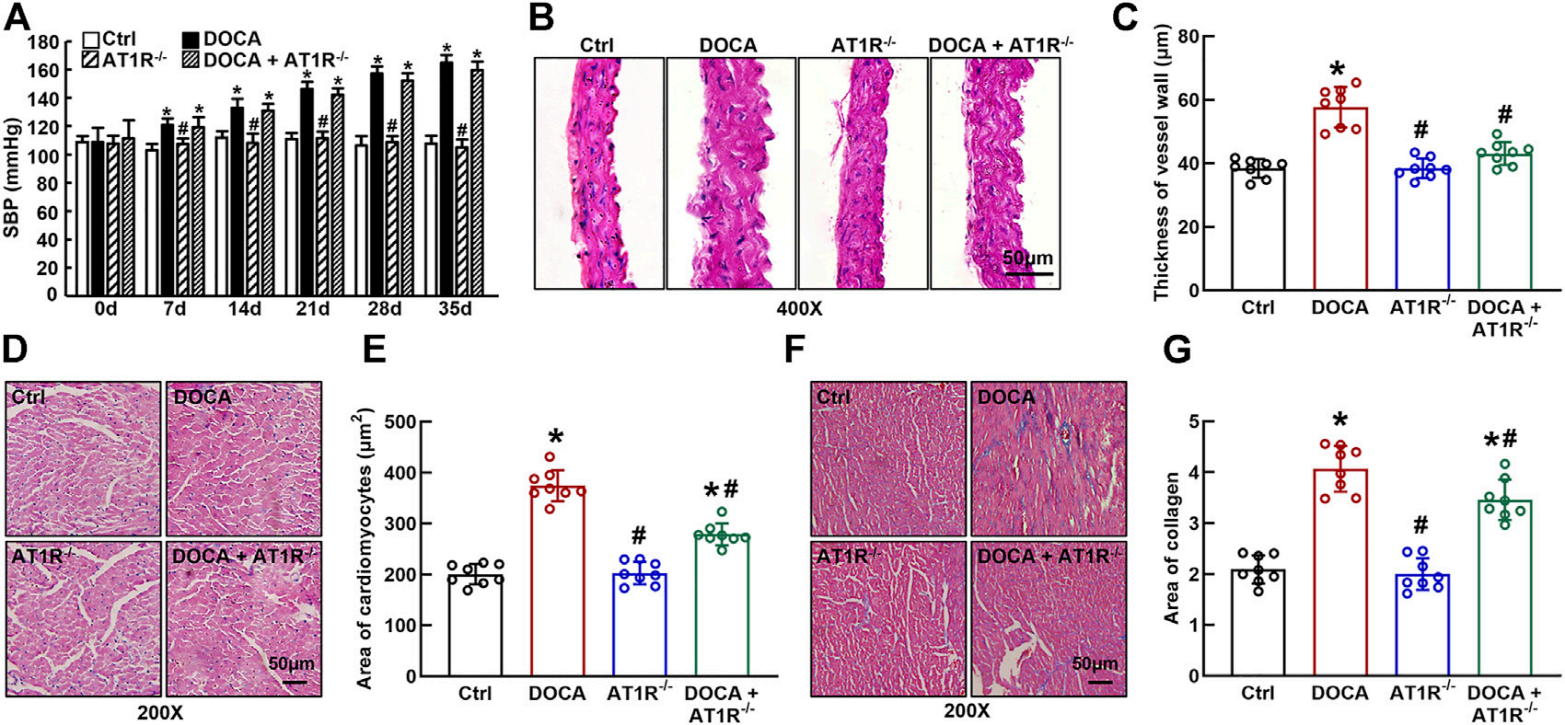
Specific myeloid AT1R KO reduced aortic and cardiac hypertrophy and cardiac fibrosis in DOCA/salt-sensitive hypertensive mice. Specific myeloid AT1R KO did not significantly affect systolic blood pressure (SBP) in DOCA/salt hypertensive mice **(A)**; Representative images of the cross section of the aortic wall **(B)** and cardiomyocyte sectional area **(D)** assessed by hematoxylin and eosin staining; quantitative analysis of aortic thickness **(C)** and cardiomyocyte sectional area **(E)**; Representative images of cardiac fibrosis assessed by Masson-trichrome staining **(F)**; Quantitative assessment of the positive collage-staining area in the heart **(G)**. Statistical comparisons were carried out with two-way ANOVA repeated measurements **(A)** or two-way ANOVA **(B–G)** followed by the Tukey post hoc test. Data were expressed as mean ± SEM. N = 8, *p < 0.05 vs. control mice, #*p* < 0.05 vs. DOCA mice.

SBP was significantly increased in DOCA mice (165 ± 2 mmHg vs. 108 ± 2 mmHg in control) compared to control mice. Specific MyeAT1R^−/-^ did not significantly affect SBP in DOCA mice (Figure 7A). Hematoxylin and eosin (HE) staining revealed that aortic wall thickness (Figures 7B and C) and cardiomyocyte sectional area (Figures 7D and E) were significantly increased in DOCA hypertensive mice. MyeAT1R^−/-^ significantly reduced aortic and cardiac hypertrophy in DOCA/salt mice. Masson-trichrome staining showed that DOCA mice had more positive collagen-stained areas than control mice. MyeAT1R^−/-^ reduced myocardial fibrosis in hypertensive DOCA mice (Figures 7F and G).

### *MyeAT1R* Gene Deficiency Reduces Aortic ROS Production and Improves Endothelial Function in DOCA SS Hypertensive Mice

**FIGURE 8:**
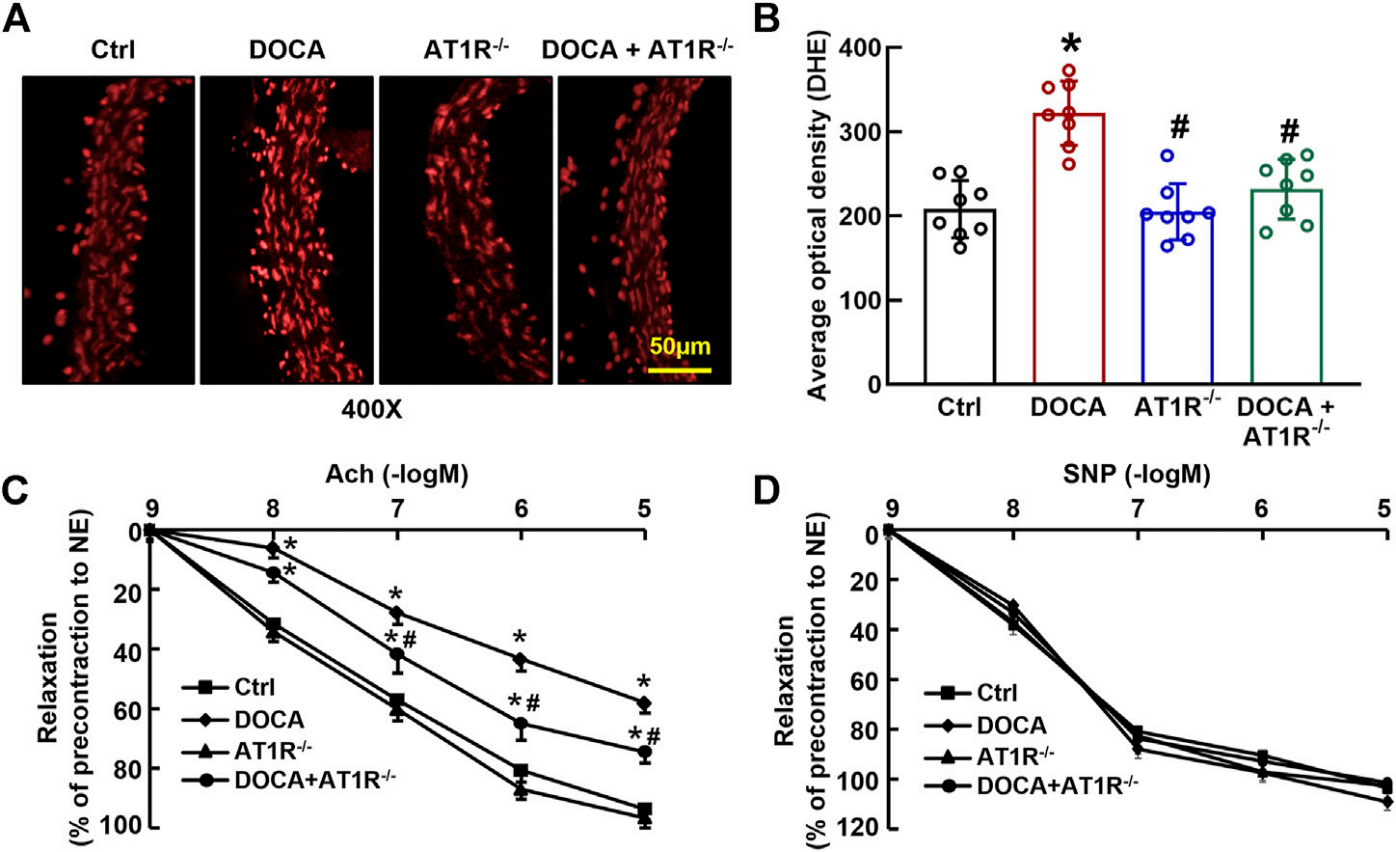
Myeloid AT1R gene deficiency reduced aortic ROS production and improved endothelial function in DOCA/salt hypertensive mice. Representative images of ROS fluorescence intensity stained by hydroethidine **(A)**; quantitative analysis of aortic ROS production **(B)**; endothelium-dependent relaxation to acetylcholine **(C)**; endothelium-independent relaxation to sodium nitroprusside **(D)**. Statistical comparisons were carried out with two-way ANOVA **(B)** or two-way ANOVA repeated measurements **(C and D)** followed by the Tukey post hoc test. Data were expressed as mean ± SEM. N = 8, *p < 0.05 vs. control mice, #*p* < 0.05 vs. DOCA mice.

We determined aortic O_2_^−^ production *in situ* using fluorescent dye DHE staining and confocal fluorescence microscopy. Oxidative fluorescence intensities were significantly increased in the aorta of DOCA hypertensive mice. MyeAT1R gene deficiency significantly reduced aortic oxidative fluorescence intensities (Figures 8A and B). Consistent with our previous findings (Cai et al., 2020), EDR to acetylcholine was significantly impaired in DOCA hypertensive mice (Emax: 64.9% ± 2.4% vs. 96.1% ± 1.6% in control mice, *p* < 0.05; ED_50_: 6.78 ± 0.13 vs. 7.35 ± 0.16 −log M in control mice, *p* < 0.05). MyeAT1R gene deficiency improved EDR to acetylcholine (Emax: 78.5% ± 1.8% vs. 64.9% ± 2.4% in DOCA mice, *p* < 0.05; ED_50_: 7.09 ± 0.19 vs. 6.78 ± 0.13 −log M in DOCA mice, *p* < 0.05, Figure 8C). There was no significant difference in endothelium-independent relaxation to sodium nitroprusside among all group mice (Figure 8D).

### *MyeAT1R* Gene Deficiency Reduced Macrophage Infiltration and Vascular Inflammation in DOCA SS Hypertensive Mice

**FIGURE 9:**
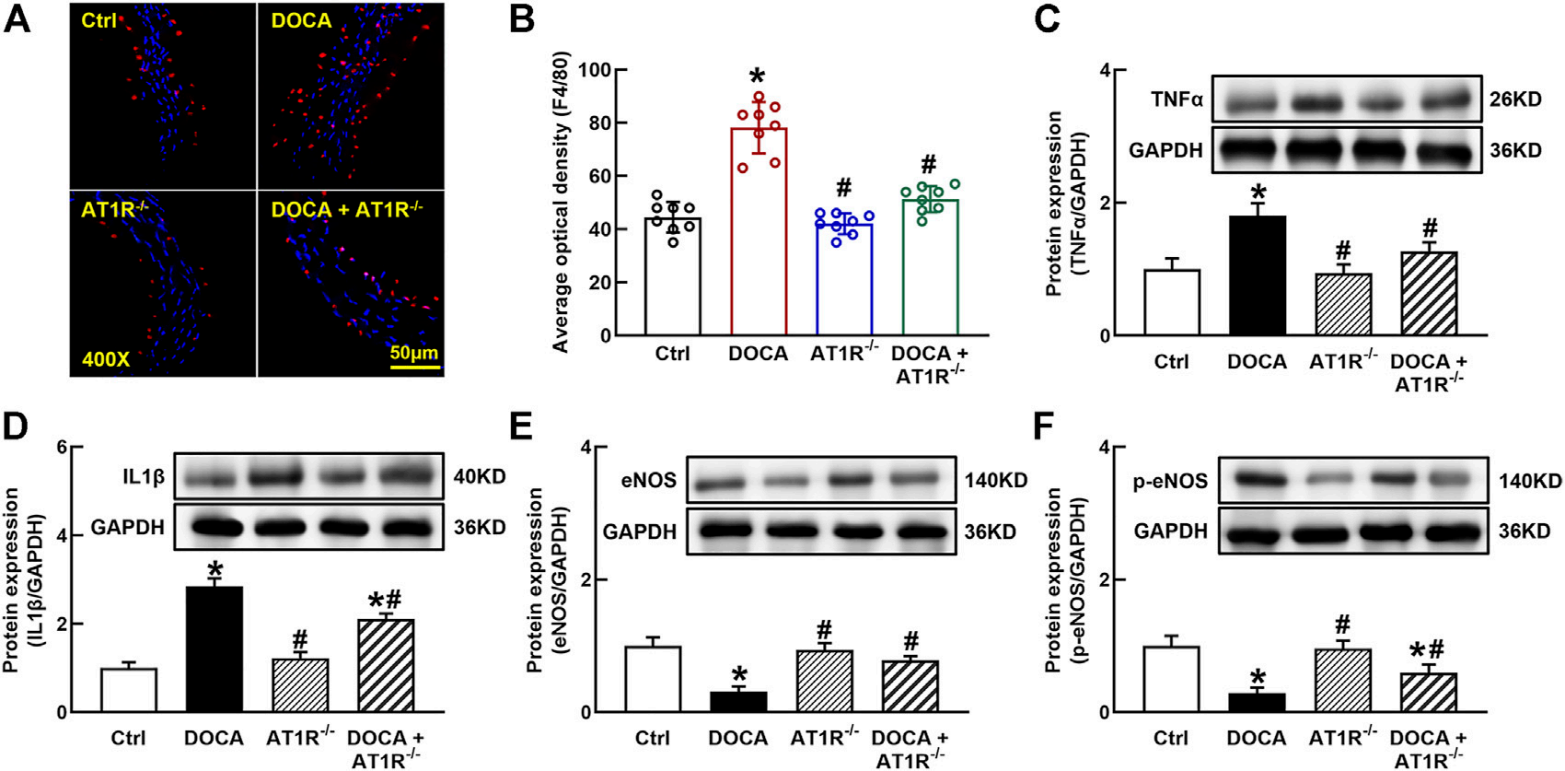
Myeloid *AT1R* gene deficiency reduced macrophage infiltration and vascular inflammation in DOCA/salt hypertensive mice. Representative images of aortic F4/80 expression assessed by immunofluorescence staining **(A)**; quantitative analysis of average immunofluorescence intensity of F4/80 expression **(B)**; protein expressions of proinflammatory cytokines, TNFα **(C)** and IL-1β **(D)**, in the aorta; protein expression of eNOS **(E)** and p-eNOS [Ser 1177, **(F)**] in the aorta. Statistical comparisons were carried out with two-way ANOVA followed by the Tukey post hoc test. Data were expressed as mean ± SEM. N = 8, *p < 0.05 vs. control mice. ^**#**^ *p* < 0.05 vs. DOCA group.

F4/80 is a molecular marker of monocytes/macrophages in mice, and we determined aortic F4/80 expression by immunofluorescence. The number of F4/80 positive cells was significantly increased in the aorta of DOCA mice, which was reduced in MyeAT1^−/−^ mice (Figures 9A and B). The protein expressions of proinflammatory cytokines TNFα and IL-1β were significantly increased in the aorta of DOCA hypertensive mice. MyeAT1R KO reduced TNFα and IL-1β expressions in DOCA hypertensive mice (Figures 9C and D). In addition, we determined aortic eNOS and p-eNOS expressions. DOCA mice had low expressions of eNOS and p-eNOS (Ser1177), which were increased in DOCA/AT1R^−/-^ mice (Figures 9E and F).

## Discussion

There is increasing evidence that inflammation and immune cell dysfunction is associated with hypertension (Guzik and Touyz, 2017; Xiao and Harrison, 2020). Ang II is an important proinflammatory mediator that promotes the accumulation of macrophages to induce vascular inflammation (Montezano et al., 2014). In the present study, we demonstrate that Ang II induces macrophage M1 polarization *via* activating AT1R, and silenced AT1R prevents Ang II-induced macrophage M1 polarization and leads to the conversion of macrophages from M1 phenotype to M2 phenotype. Furthermore, we show that Ang II/AT1R-induced M1 polarization is mediated by the activation of the HIF1α/TLR4/NF*κ*B pathway. These results suggest that Ang II/AT1R controls macrophage phenotype and polarization. Using the mice with specific knockout of myeloid AT1R, we demonstrate that AT1R deficiency in myeloid cells inhibits macrophage HIFα/NF*κ*B activation and protects against endothelial dysfunction and cardiovascular injury without significantly reducing SBP in DOCA SS mice.

Macrophage heterogeneity and polarization are considered important features of diverse diseases, including infection, obesity, hypertension, and cardiovascular diseases (Lumeng et al., 2007; Harwani, 2018). Macrophages can modulate their phenotypes in response to environmental signals. M1 and M2 macrophages represent the extreme states of macrophage polarization. At present, the molecular mechanisms that control M1/M2 polarization are still not fully understood. In general, classically activated M1 macrophages are induced by LPS or Th1 cytokines (Martinez et al., 2008). M1 macrophages increase the production of ROS and proinflammatory cytokines, which helps to enhance the antimicrobial ability and the control of the infection (Chen et al., 2018). However, the inappropriate activation of macrophages may cause chronic low-grade inflammation, resulting in tissue and cell damage (Oishi and Manabe, 2018). Macrophage activation in the infected tissues is usually triggered by foreign antigens or pathogens (Ma et al., 2003). The present study demonstrates that Ang II, an endogenous hormone, can induce macrophage M1 polarization and activation *via* stimulating AT1R because the genetic manipulation of AT1R expression can modify Ang II-induced changes in macrophage phenotype and polarization.

Macrophage activation is often associated with a hypoxic environment in infected tissues, and hypoxia can profoundly affect macrophage functions (Díaz-Bulnes et al., 2019). HIF1α is a transcriptional factor which is essential for the regulation of macrophage function (Nakayama et al., 2013). In normal oxygen conditions, HIF1α is rapidly degraded by PHD, and hypoxia stabilizes and activates HIF1α by the inhibition of PHD-dependent degradation of the HIF1α subunit (Zhu et al., 2014; Pezzuto and Carico, 2018). In addition to hypoxia, various other factors, such as ROS and transition metals, can also regulate HIF1α activity (Chun et al., 2002; Ullah et al., 2017). It is well known that foreign antigen induces macrophage activation *via* stimulating TLR4/NF*κ*B pathway (Zhang et al., 2015; He et al., 2019). Recent studies have shown there is an interaction between HIF1α and TLR4/NF*κ*B signaling pathway, which induces macrophage activation (Rius et al., 2008). The present study demonstrates that Ang II activates HIF1α *via* the inhibition of PHD-dependent degradation of HIF1α, which further activates the TLR4/NF*κ*B pathway. In addition, antioxidants prevent Ang II downregulation of PHD2 and upregulation of HIF1α and NF*κ*B activation. Therefore, these results suggest that Ang II/AT1R induces macrophage activation *via* ROS-mediated HIF1α/TLR4/NF*κ*B pathway.

Sustained accumulation of macrophages in the vascular wall and kidney is considered one of the important mechanisms for hypertension, particularly for Ang II-induced hypertension and SS hypertension (Thang et al., 2015; Huang et al., 2018; Tian et al., 2021). Our research team has long been committed to the mechanisms of vascular injury in SS hypertension. We have previously shown that although plasma renin level is low, vascular injury in SS hypertension is related to the functional upregulation of local Ang II (Zhou et al., 2003; 2006), and treatment with ARB candesartan can improve endothelial function and vascular insulin resistance and reduce vascular inflammation and oxidative stress (Zhou et al., 2009). Furthermore, we demonstrate the chemical depletion of macrophages can mimic vascular beneficial effects of ARB in SS hypertension (Tan et al., 2017). These results suggest that both AT1R and macrophages play an important role in the pathogenesis of SS hypertension. To identify the role of macrophage AT1R in the vascular injury of SS hypertension, we crossed AT1R^loxp/loxp^ mice with LysM-Cre mice to generate the mice with specific deletion of myeloid AT1R. The AT1R expression is significantly increased in the peritoneal macrophage of DOCA SS hypertensive mice. Consistent with our findings in *in vitro*, the deletion of myeloid AT1R *in vivo* inhibits macrophage HIF1α/NF*κ*B activation. In addition, the depletion of myeloid AT1R provides vascular beneficial effects, including improving endothelial function and reducing vascular oxidative stress, vascular inflammation, and injury in DOCA SS hypertensive mice. The vascular beneficial effects of AT1R KO cannot be explained by hemodynamics because blood pressure is not reduced in DOCA/AT1R^−/-^ mice. These results suggest that myeloid AT1R plays an important role in the vascular injury of SS hypertension. Because DOCA hypertension is a low plasma renin model (Li et al., 2003), the interpretation of the results should be cautious. We speculate the following: 1) despite the low plasma renin level, DOCA mice may upregulate macrophage local RAS, such as the upregulation of AT1R, which is similar to our previous findings in hypertensive DS rats (Zhou et al., 2003; Zhou et al., 2006) 2) the results from the present study may apply to DOCA hypertension or SS hypertension, and whether the results can apply to other hypertension remains to be confirmed.

There are several studies *in vivo* that macrophage AT1R can affect macrophage phenotype and vascular and renal homeostasis. Consistent with our findings that macrophages play a deleterious role, Yamamoto et al. (2011) reported that the transplantation of bone marrow from AT1R^−/-^ apoE^−/-^ mice to apoE^−/-^ reduced macrophage M1 cytokines and renal injury-induced acceleration of atherosclerosis. Wang et al. (2021) showed that Ang II stimulation of AT1R could induce M1 macrophage polarization and macrophage infiltration *via* stimulating the YAP pathway, and blockade of AT1R by ARB could reduce aortic inflammation and aortic dissection incidence. Zhang et al. (2014) reported somewhat opposite effects that depletion of macrophage AT1A receptor (a subtype of AT1R) heightened M1 macrophage proinflammatory cytokines and exacerbated renal fibrosis induced by unilateral ureteral obstruction. They further demonstrated that the augmentation of renal fibrosis in macrophage AT1A receptor–deficiency mice is mediated by the stimulation of the renal IL-1 receptor. These results suggest that macrophage AT1R plays a variety of roles in hypertension and cardiovascular and renal diseases, which may be related to AT1R subtypes, organ specificity and disease conditions.

Limitations: The present study has several limitations. First, we did not detect a difference in blood pressure between DOCA mice and DOCA/AT1R^−/-^ mice. We used the tail-cuff method to measure blood pressure, which may not accurately measure a slight change in blood pressure. However, we believe that the result is reliable because the result is consistent with our previous findings in hypertensive DS rats, which show that ARB candesartan does not significantly reduce blood pressure (Zhou et al., 2009). Next, LyzM-Cre can target other immune cells, such as lymphocytes or dendritic cells (Jones et al., 2016), thus, it cannot be completely ruled out that vascular beneficial effects in myeloid AT1R deficiency mice may also be partly due to the depletion of other immune cells, which is worthy of study in the future. Third, in the present study, we used THP1/macrophage, which is widely used in the field of macrophage biology, because it can overcome the issues of limited lifespan and inter-individual variability that affects human monocyte-derived macrophages (Tedesco et al., 2018). However, there are still some arguments about whether THP1-derived macrophages can entirely mimic human macrophages (Tedesco et al., 2015, 2018). In the present study, the results from THP1 macrophage *in vitro* and mouse peritoneal macrophages *ex vivo* are consistent to show that AT1R can activate macrophage HIF1α/NF*κ*B pathway to induce macrophage activation. Finally, the present study determined endothelium-dependent relaxation to acetylcholine and p-eNOS expression. However, p-eNOS expression may not actually reflect that eNOS functions to produce NO in some pathological conditions because it has been reported that there is an uncoupling eNOS which may produce superoxide anion instead of NO in salt-sensitive hypertension (Zhou et al., 2003; Wu et al., 2021). Thus, a direct measurement of aortic NO production or eNOS activity may further corroborate the role of endothelium-derived NO in myeAT1R^−/-^ mice.

In summary, the present study demonstrates that Ang II controls macrophage phenotype and function *via* direct stimulation of AT1R in macrophages, Ang II/AT1R-induced macrophage activation is mediated by HIF1α/TLR4/NF*κ*B pathway, and the depletion of myeloid AT1R can prevent macrophage activation and provide vascular beneficial effects in SS hypertension. Therefore, the present study reveals an unmasked finding that the dysfunction of AT1R in macrophages may cause an abnormal immune response in macrophages, which may in part be the mechanism of vascular injury in SS hypertension.

## Data Availability

The original contributions presented in the study are included in the article/Supplementary Materials, further inquiries can be directed to the corresponding authors.
